# Multi-Condition Cultivation Reveals the Host Plant-Dependent Gut Bacteria Diversity in Tomato Leafminer (*Tuta absoluta*) Larvae

**DOI:** 10.3390/insects17010081

**Published:** 2026-01-10

**Authors:** Xiaoyu Fang, Ruoyi Wen, Liyan Yang, Jianyang Guo, Wenjun Shen, Nianwan Yang, Fanghao Wan, Zhichuang Lü, Wanxue Liu

**Affiliations:** 1 College of Life Science, Shanxi Normal University, Taiyuan 030031, China; 13068081963@163.com (X.F.); yangswallow163@163.com (L.Y.); 2 State Key Laboratory for Biology of Plant Diseases and Insect Pests, Key Laboratory for Prevention and Control of Invasive Alien Species of Ministry of Agriculture and Rural Affairs, Institute of Plant Protection, Chinese Academy of Agricultural Sciences, Beijing 100193, China; 18175197411@163.com (R.W.); guojianyang@caas.cn (J.G.); shenwenjun@caas.cn (W.S.); yangnianwan@caas.cn (N.Y.); wanfanghao@caas.cn (F.W.); 3 Western Agricultural Research Center, Chinese Academy of Agricultural Sciences, Changji 831100, China

**Keywords:** *Tuta absoluta*, tomato, eggplant, traditional culture methods, gut microbiota

## Abstract

Tomato leafminer (*Tuta absoluta*) significantly affects tomato (*Solanum lycopersicum*) and eggplant (*Solanum melongena*) crops worldwide, with its feeding patterns being closely associated with its gut microbiota. We aimed to compare the cultivable gut bacteria of *T. absoluta* larvae fed on tomato and eggplant to investigate their role in host adaptation. Gut bacteria were cultivated on Luria–Bertani broth, nutrient agar, and Brain Heart Infusion media under different temperature conditions, followed by morphology- and 16S rRNA-based identification. Notably, both feeding groups revealed distinct gut bacterial community structures. Tomato-fed larvae harbored bacteria spanning eight species, five genera, four families, and two phyla. In contrast, eggplant-fed larvae exhibited greater microbial diversity, encompassing 15 species, 10 genera, 9 families, and 3 phyla, including unique genera such as *Pseudomonas* and *Pectobacterium*, which was attributed to the host plant contribution. *Enterococcus mundtii* was the most dominant bacterium, and species such as *Bacillus wiedmannii* and *Micrococcus luteus* were most thermotolerant. Overall, these findings highlight the importance of multi-condition culture approaches for thoroughly characterizing insect gut microbiota and underscore the role of host plants in pest adaptability by modulating gut microbial communities, providing new insights for developing sustainable control strategies utilizing “plant–insect–microorganism” interactions.

## Simple Summary

The tomato leafminer *Tuta absoluta* is a major global pest of tomato (*Solanum lycopersicum*) and eggplant (*Solanum melongena*). Although feeding on different host plants is known to influence its gut microbiota, comprehensive research remains lacking. Hence, we compared the cultivable gut bacteria in tomato- and eggplant-fed *T. absoluta* larvae to investigate their role in host adaptation. Herein, the microbes were traditionally cultivated on Luria–Bertani broth, nutrient agar, and Brain Heart Infusion media under different temperature conditions, followed by microbial identification based on morphology and 16S rRNA sequences. Distinct gut bacterial community structures were found between the two host-feeding groups, with eggplant-fed larvae exhibiting higher microbial diversity. Host plant species significantly influenced the composition and diversity of the gut bacteria. *Enterococcus mundtii* exhibited the highest abundance under most conditions, whereas high-temperature conditions enriched thermotolerant strains such as *Bacillus wiedmannii* and *Micrococcus luteus*. Altogether, these findings underscore the significance of a multi-condition culture strategy for comprehensive analysis of insect gut microbiota regarding pest adaptability to different plants. Future research needs to focus on functional validation of the isolated bacterial strains to elucidate the underlying “plant–insect–microorganism” interactions to provide novel insights for developing sustainable control strategies.

## 1. Introduction

The tomato leaf miner *Tuta absoluta* (Meyrick) (Lepidoptera: Gelechiidae) is a devastating pest of the tomato (*Solanum lycopersicum*) plant worldwide [1,2], with its larvae mining leaves, stems, and fruits, thereby leading to damages of up to 80–100% yield loss in severe cases [2]. Although it also attacks other solanaceous crops, such as potato, eggplant (*Solanum melongena*), and tobacco [3], its invasiveness and damage are more severe on tomato plants. However, the internal mechanisms underlying host adaptability remain unelucidated [4,5]. Reportedly, different host plants significantly affect the growth and physiology of *T. absoluta* [6]. For example, tomato-reared larvae exhibit superior growth and development compared to those reared on eggplant [7], with such adaptive differences closely attributed to the physiological responses of the host plants. *T. absoluta* can trigger distinct defense responses in tomatoes and eggplants. For instance, tomato leaves exhibit lower levels of defense compounds and weaker responses in insect-resistant pathways than eggplant leaves [4]. In addition to affecting the plants themselves, host plant differences directly influence the intestinal microenvironment of the insects [8,9]. Sequencing results have shown significant differences in the gut microbiota structure and diversity of larvae fed on different hosts (e.g., tomato, eggplant, and potato) [6,10]. These findings closely associate host plant identity with insect adaptability, with gut microbiota playing a pivotal role.

In the plant–insect–gut microbiota interaction, gut microorganisms act as key mediators influencing insect adaptability and feeding preferences. They participate in essential physiological processes, including growth and development, nutrient metabolism, immune regulation, and detoxification of plant secondary metabolites [11,12,13]. For example, in *Drosophila* species that feed on rotten fruits, fermentative bacteria such as acetic acid and lactic acid bacteria promote development by modulating the host insulin signaling pathway; their removal produces viable but stunted and slow-growing adults [14]. Gut microbes also contribute to detoxification and can assist in degrading plant defense compounds [15]. For example, *Pseudomonas fulva* ZJU1 in the silkworm gut metabolizes 1-deoxynojirimycin (DNJ) from mulberry leaves to neutralize the plant’s chemical defense [16]. When insects adapt to different host plants, their gut microbiota structure shifts accordingly to adapt to the new nutritional and chemical environment [11,14,17,18,19]. This host-driven restructuring has been observed across multiple insect species. For instance, the gut microbiota composition of *Spodoptera frugiperda* varies with its host plant [20], whereas in *Plutella xylostella*, switching from radish seedlings to pea plants over successive generations can lead to reduced gut microbial diversity and altered community composition [21]. Collectively, these findings demonstrate that insects adapt to different plant nutritional niches by modulating their gut microbes. The same principle applies to *T. absoluta*, where 16S rRNA sequencing has confirmed significant differences in gut bacterial structure and diversity between larvae fed on tomato and those fed on eggplant [22,23]. However, research on *T. absoluta* remains largely reliant on sequencing analyses, with the lack of systematic isolation and identification of its culturable gut bacteria limiting further understanding of their specific functions.

Obtaining pure bacterial cultures is essential for elucidating the functional roles of insect gut microbiota. Although culture-independent methods such as 16S rRNA gene sequencing and metagenomics have greatly advanced knowledge of gut microbial diversity [24,25,26,27], they mainly elucidate community composition and predicted gene functions, rarely yielding culturable isolates needed for physiological, biochemical, and functional validation. Consequently, traditional culture-based approaches offer irreplaceable advantages, as they allow for the adjustment of key parameters, including temperature, pH, and specific nutrients, to closely mimic the insect intestinal microenvironment. This simulation facilitates the effective isolation and cultivation of diverse microbes, which, in turn, provides the essential basis for investigating their specific functions [28,29]. Building on this, we aimed to identify cultivable gut bacteria of *T. absoluta* larvae fed on tomato and eggplant to investigate their role in host adaptation. Herein, three nutritionally distinct yet versatile media, namely Luria–Bertani (LB), nutrient agar (NA), and Brain Heart Infusion (BHI), were employed to recover a broad range of bacteria with diverse nutrient requirements [19]. Isolations were performed at three incubation temperatures (25 °C, 31 °C, and 37 °C), considering 25 °C approximates the insect gut environment, 31 °C favors mesophilic bacteria, and 37 °C promotes the growth of thermotolerant or specialized strains. This multi-condition strategy aimed to achieve comprehensive recovery of the cultivable gut bacterial community.

Additionally, to clarify the differences in gut bacterial composition of *T. absoluta* after feeding on different host plants (tomato versus eggplant) and to isolate functionally relevant strains, the gut bacteria of cultivated larvae were isolated and purified using traditional methods with the three selected media and temperature conditions. The isolates were subsequently identified based on morphological characteristics and molecular analyses. Overall, this study provides a foundational collection of culturable bacterial strains to support future investigations into the functional roles of gut microbiota in the host adaptation of *T. absoluta*.

## 2. Materials and Methods

### 2.1. Insects and Plants

Tomato (*S. lycopersicum* “Dafen”) and eggplant (*S. melongena* “Zilong”) were used as host plants in this study. They were grown in a mixture of nutrient-rich soil and vermiculite (2:1, *v*/*v*) in a plant growth chamber at the Institute of Plant Protection, Chinese Academy of Agricultural Sciences, Beijing. The original population of *T. absoluta* was collected from Yuxi, Yunnan Province, in August 2018 and was periodically supplemented with field-collected individuals. All insects were maintained at 25 ± 2 °C, 50–60% relative humidity, and a 14L:10D photoperiod. A stable population was established by continuously rearing insects on their respective host plants for three generations, and third-instar larvae from the third generation were used for subsequent microbial analyses.

### 2.2. Gut Dissection

The healthy third-instar larvae fed on tomatoes or eggplants were assigned to three replicate groups per host plant, each containing 50 larvae. The larvae were starved for 8 h before dissection. All dissection tools were sterilized in an autoclave for 30 min. First, the larvae were rinsed twice with sterile water (1 min each) for surface-sterilization, followed by immersion in 75% ethanol for 20–30 s, and two final rinses with sterile water (1 min each). Following this, surface-sterilized larvae were transferred to a sterile Petri dish, and their gut was carefully extracted from the posterior end using sterile forceps. Each gut was placed in a 1.5 mL sterile centrifuge tube containing 50 μL of sterile phosphate-buffered saline (Beijing Solarbio Science & Technology Co., Ltd., Beijing, China) and homogenized [30,31].

### 2.3. Isolation and Purification of Culturable Gut Bacteria

The extracted intestinal homogenate was serially diluted to concentrations ranging from 1.0 × 10^−1^ to 1.0 × 10^−6^. Next, a 100-μL aliquot of each dilution was spread onto LB, NA, and BHI solid media, with three replicate plates prepared for each media and dilution. All plates were incubated at 25 °C, 31 °C, and 37 °C for 24–48 h, following which, the number of colonies with different colors, shapes, and sizes was recorded. Distinct single colonies with varying morphologies were isolated and purified 2–5 times on their respective media, followed by the inoculation of a single purified colony into the liquid medium for culture in a shaker at 180 rpm for 24–48 h to obtain the bacteria. Purified strains were stored in glycerol tubes at −80 °C for future use.

### 2.4. Identification of Culturable Gut Bacteria

#### 2.4.1. Morphological Identification

Regarding morphological analysis, the isolated and purified single colonies were observed for the shape, color, luster, transparency, and elevation with reference to *Bergey’s Manual of Systematic Bacteriology* [32]. The isolated strains were subjected to Gram staining, and cell morphology and Gram reaction (positive or negative) were observed using an oil-immersion lens.

#### 2.4.2. Molecular Identification

For molecular identification, 16S rRNA gene sequencing was performed in this study. Briefly, a single colony was used to prepare the bacterial suspension, and the bacterial pellet was collected after centrifugation at 10,000× *g* for 10 min. The genomic DNA was extracted using the QIAamp DNA Microbiome Kit (Qiagen, Hilden, Germany) per the manufacturer’s instructions, which served as the polymerase chain reaction (PCR) template. Universal primers used to amplify the 16S rRNA gene were 27F (5′-AGAGTTTGATCCTGGCTCAG-3′) and 1492R (5′-CGGTTACCTTGTTACGACTT-3′). The PCR mixture (20 μL) comprised 2× Pro Taq Master Mix (10 μL), forward primer (1 μL), reverse primer (1 μL), template DNA (5 ng), and sterile double-distilled water to a final volume of 20 μL. The PCR amplification program was set as follows: pre-denaturation at 95 °C for 3 min; followed by 25 cycles of denaturation at 95 °C for 30 s, annealing at 56 °C for 30 s, and extension at 72 °C for 45 s; with a final extension at 72 °C for 10 min. Following amplification, a 5-μL aliquot of each PCR product was electrophoresed on 1% agarose gel. The products showing clear and specific bands of the expected size were then qualified and sent to Shanghai Meiji Biotechnology Co., Ltd., Shanghai, China for sequencing. The obtained sequences were submitted to the National Center for Biotechnology Information database (http://blast.ncbi.nlm.nih.gov/Blast.cgi (accessed from 26 June 2025 to 29 August 2025)) to screen for the most similar known bacterial strain sequences using the Basic Local Alignment Search Tool.

### 2.5. Phylogenetic Analysis

A phylogenetic tree was constructed using the Neighbor-Joining method and the MEGA 11 software. The target sequences were aligned using the integrated ClustalW algorithm, and multiple sequence alignments were manually refined. The reliability of the phylogenetic topology was assessed using 1000 bootstrap replications.

### 2.6. Data Analyses

All isolated pure strains were enumerated and their relative abundances were calculated to analyze the composition of the cultivable gut microbiota. Each pure bacterial colony isolated under different culture conditions (varying media and temperature) was considered an independent strain. Based on their molecular identification, the strains were grouped and counted according to their taxonomic classification and host-feeding source (tomato or eggplant). The relative abundance of a given bacterial species was calculated as follows:

Proportion of isolates (%) = (Number of strains of the same bacterial species/Total number of cultivable strains within the same host group) × 100

After calculating the relative abundances, the data were compiled into an Excel 2021 spreadsheet and plotted on a graph.

## 3. Results

### 3.1. Morphology and Culture Characteristics of Colonies

Various bacteria were isolated from the intestinal tracts of *T. absoluta* larvae fed either tomato or eggplant leaves. The colonies were isolated from three culture media (LB, NA, and BHI) incubated at three different temperatures (25 °C, 31 °C, and 37 °C) (Table 1).

**Figure 1:**
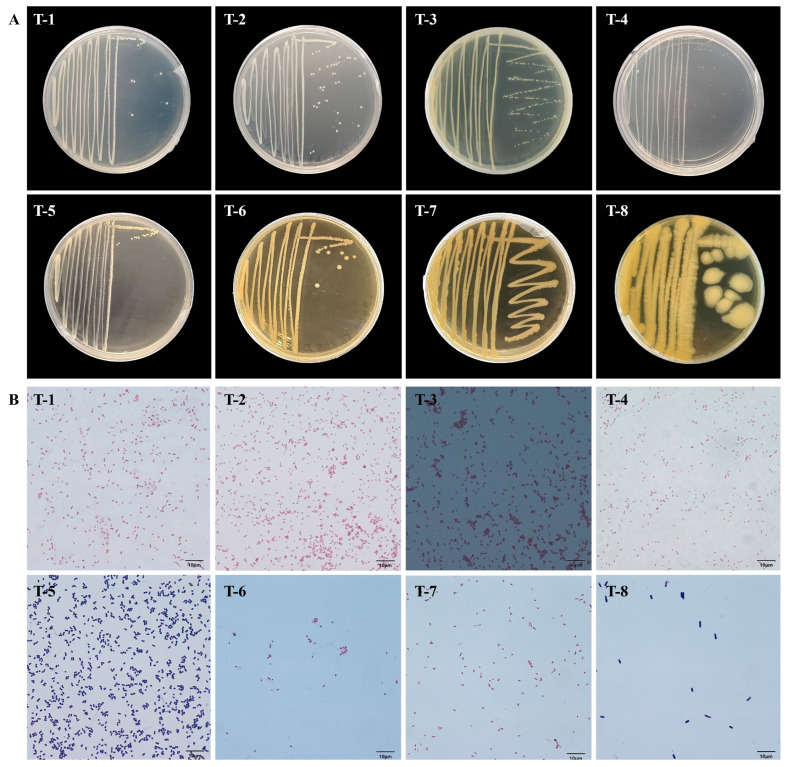
Morphological characteristics of culturable gut bacteria isolated from tomato-fed *Tuta absoluta* larvae. (**A**) Colony morphology; (**B**) Cellular morphology (Gram staining).

Representative colonies from each morphological type were selected for detailed observation. Notably, eight bacterial strains (T-1–T-8) were isolated from the gut of tomato-fed *T. absoluta* larvae, containing six Gram-negative and two Gram-positive strains, with T-1, T-2, T-4, and T-7 being short-rod; T-3, T-5, and T-6 being cocci; and T-8 being long-rod shaped (Figure 1; Table 2).

**Figure 2:**
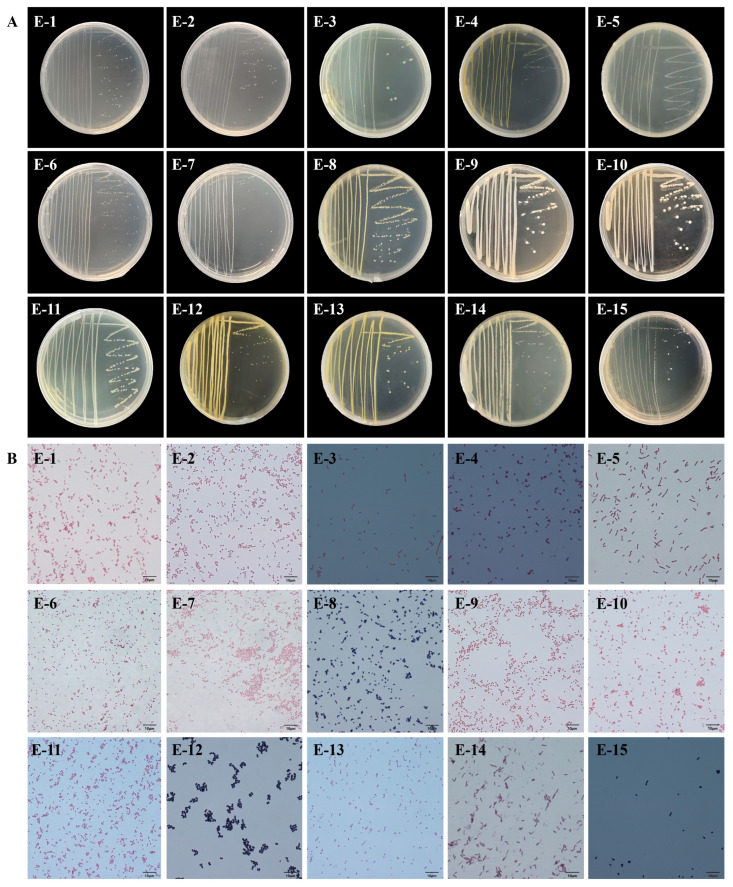
Morphological characteristics of culturable gut bacteria isolated from eggplant-fed *Tuta absoluta* larvae. (**A**) Colony morphology; (**B**) Cellular morphology (Gram staining).

From eggplant-fed larvae, 15 strains (E-1–E-15) were obtained, containing 12 Gram-negative and 3 Gram-positive strains. Morphological examination revealed that strains E-1–E-4, E-9–E-11, and E-13 were short rods; E-6–E-8, E-12, and E-15 were cocci; and E-5 and E-14 were long rods (Figure 2; Table 2).

### 3.2. Composition of Intestinal Bacteria in Tomato- and Eggplant-Feeding Populations

Based on 16S rRNA sequencing, eight bacterial strains were isolated from the gut of the tomato-feeding population, belonging to two phyla, four families, five genera, and eight species (Table 3). The isolates from the phylum Pseudomonadota (Proteobacteria) encompassed the following two families and three genera: *Enterobacter* and *Kluyvera* of Enterobacteriaceae; *Aeromonas* of Aeromonadaceae. Strains from the phylum Bacillota (Firmicutes) included two families and two genera, as follows: *Enterococcus* of Enterococcaceae; *Bacillus* of Bacillaceae.

From the eggplant-fed larvae, 15 bacterial strains, belonging to 3 phyla, 9 families, and 10 genera, were isolated (Table 3). The Pseudomonadota (Proteobacteria) included the following four families and four genera: Enterobacteriaceae (genus *Enterobacter*), Pectobacteriaceae (genus *Pectobacterium*), Erwiniaceae (genus *Pantoea*), and Pseudomonadaceae (genus *Pseudomonas*). Bacillota (Firmicutes) include the following three families and four genera: Bacillaceae (genera *Mesobacillus* and *Bacillus*), Enterococcaceae (genus *Enterococcus*), and Staphylococcaceae (genus *Mammaliicoccus*). Lastly, Actinomycetota (Actinobacteria) includes two families and two genera, as follows: Micrococcaceae (genus *Micrococcus*); Dermabacteraceae (genus *Brachybacterium*).

### 3.3. Phylogenetic Analysis of Culturable Intestinal Bacteria from T. absoluta Fed on Tomato and Eggplant

**Figure 3:**
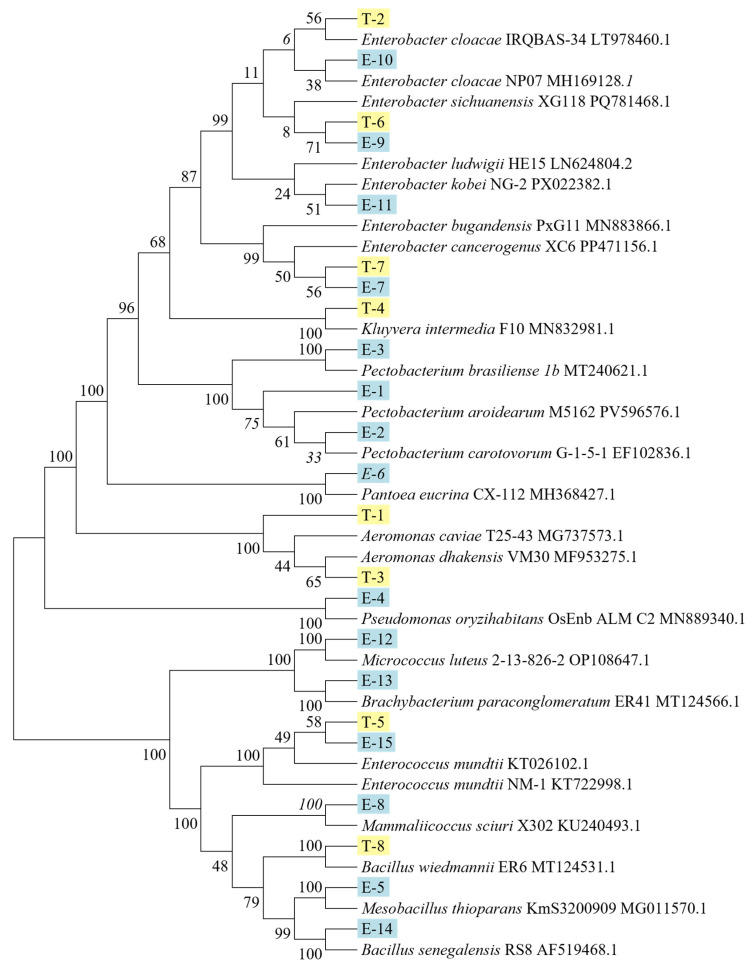
Phylogenetic tree of isolated strains and related reference strains based on 16S rRNA gene sequences; yellow represents cultivable strains isolated from *Tuta absoluta* feeding on tomato, and blue represents cultivable strains isolated from *Tuta absoluta* feeding on eggplant.

A phylogenetic tree was constructed based on the 16S rRNA sequences of the culturable gut bacteria, revealing three major clades (Figure 3).

Clade I comprising Pseudomonadota (Proteobacteria), containing several monophyletic groups, including strains T-2, E-10, T-6, E-9, E-11, T-7, and E-7 (*Enterobacter*) clustered with reference strains of *Enterobacter cloacae* and *Enterobacter sichuanensis*; strain T-4 (*Kluyvera*) clustered with *Kluyvera intermedia*; strains E-1, E-2, and E-3 (*Pectobacterium*) formed a cluster with *Pectobacterium brasiliense*; strain E-6 (*Pantoea*) clustered with *Pantoea eucrina*; strains T-1 and T-3 (*Aeromonas*) grouped with *Aeromonas caviae*; and strain E-4 (*Pseudomonas*) clustered with *Pseudomonas oryzihabitans*.

Clade II comprised Actinomycetota (Actinobacteria), including strains E-12 (*Micrococcus*), which clustered with *Micrococcus luteus*, and E-13 (*Brachybacterium*), which clustered with *Brachybacterium paraconglomeratum*.

Lastly, Clade III comprised Bacillota (Firmicutes), including strains T-5 and E-15 (*Enterococcus*) clustered with *Enterococcus mundtii*; strain E-8 (*Mammaliicoccus*) clustered with *Mammaliicoccus sciuri*; strain T-8 (*Bacillus*) grouped with *Bacillus wiedmannii* and strain E-14 (*Bacillus*) grouped with *Bacillus senegalensis*; and strain E-5 (*Mesobacillus*) clustered with *Mesobacillus thioparans*.

### 3.4. Variation in Culturable Gut Bacterial Composition of T. absoluta Under Different Culture Conditions

**Figure 4:**
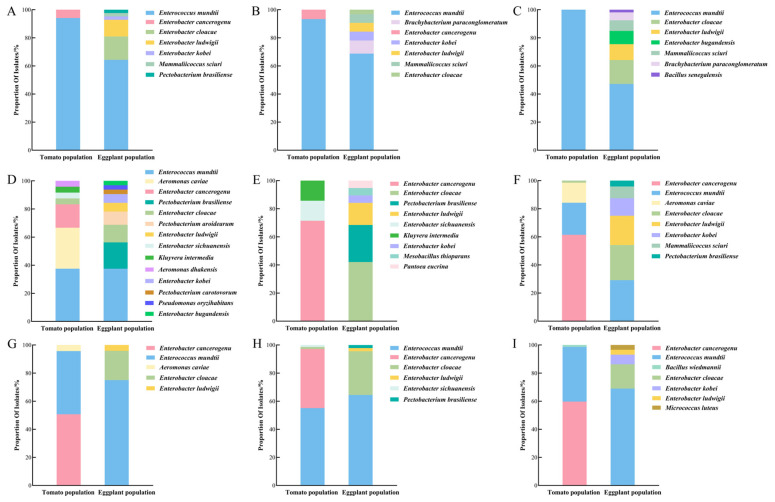
Relative abundance of culturable gut bacteria at the species level from *Tuta absoluta* larvae fed on tomato or eggplant, under different culture conditions: (**A**) Luria–Bertani (LB) medium, 25 °C; (**B**) LB medium, 31 °C; (**C**) LB medium, 37 °C; (**D**) nutrient agar (NA) medium, 25 °C; (**E**) NA medium, 31 °C; (**F**) NA medium, 37 °C; (**G**) Brain Heart Infusion (BHI) medium, 25 °C; (**H**) BHI medium, 31 °C; (**I**) BHI medium, 37 °C.

On LB medium, at 25 °C (Figure 4A), the following two bacterial species were isolated from tomato-fed larvae: *E. mundtii* (94.12%) and *Enterobacter cancerogenus* (5.88%). In contrast, six species were obtained from the eggplant-fed larvae, namely *E. mundtii* (64.29%), *E. cloacae* (16.67%), *Enterobacter ludwigii* (11.9%), *Enterobacter kobei* (2.38%), *M. sciuri* (2.38%), and *P. brasiliense* (2.38%). At 31 °C (Figure 4B), tomato-fed larvae yielded the following two species: *E. mundtii* (93.33%) and *E. cancerogenus* (6.67%), whereas eggplant-fed larvae yielded six species, namely *E. mundtii* (68.75%), *B. paraconglomeratum* (9.38%), *E. kobei* (6.25%), *E. ludwigii* (6.25%), *M. sciuri* (6.25%), and *E. cloacae* (3.13%). At 37 °C (Figure 4C), only *E. mundtii* (100%) was isolated from tomato-fed larvae, whereas eggplant-fed larvae yielded the following seven species: *E. mundtii* (47.17%), *E. cloacae* (16.98%), *E. ludwigii* (11.32%), *Enterobacter bugandensis* (9.43%), *M. sciuri* (7.55%), *B. paraconglomeratum* (5.66%), and *B. senegalensis* (1.89%).

On NA medium, at 25 °C (Figure 4D), the following seven species were isolated from tomato-fed larvae: *E. mundtii* (37.50%), *A. caviae* (29.17%), *E. cancerogenus* (16.67%), *E. cloacae* (4.17%), *E. sichuanensis* (4.17%), *K. intermedia* (4.17%), and *Aeromonas dhakensis* (4.17%). In total, nine species were obtained from eggplant-fed larvae: *E. mundtii* (37.50%), *P. brasiliense* (18.75%), *E. cloacae* (12.51%), *Pectobacterium aroidearum* (9.38%), *E. ludwigii* (6.25%), *E. kobei* (6.25%), *Pectobacterium carotovorum* (3.13%), *P. oryzihabitans* (3.13%), and *E. bugandensis* (3.13%). At 31 °C (Figure 4E), tomato-fed larvae yielded three species: *E. cancerogenus* (71.43%), *E. sichuanensis* (14.29%), and *K. intermedia* (14.29%). In contrast, eggplant-fed larvae yielded six species: *E. cloacae* (42.11%), *P. brasiliense* (26.32%), *E. ludwigii* (15.79%), *E. kobei* (5.26%), *M. thioparans* (5.26%), and *P. eucrina* (5.26%). At 37 °C (Figure 4F), the following four species were isolated from tomato-fed larvae: *E. cancerogenus* (61.43%), *E. mundtii* (22.86%), *A. caviae* (14.29%), and *E. cloacae* (1.43%). In contrast, eggplant-fed larvae yielded six species: *E. mundtii* (29.17%), *E. cloacae* (25.00%), *E. ludwigii* (20.83%), *E. kobei* (12.50%), *M. sciuri* (8.33%) and *P. brasiliense* (4.17%).

On BHI medium, at 25 °C (Figure 4G), three species each were obtained from tomato-fed larvae—*E. cancerogenus* (50.72%), *E. mundtii* (44.93%), and *A. caviae* (4.35%)—and eggplant-fed larvae—*E. mundtii* (75.00%), *E. cloacae* (20.84%), and *E. ludwigii* (4.17%). At 31 °C (Figure 4H), four species each were obtained from tomato-fed larvae—*E. mundtii* (55.07%), *E. cancerogenus* (42.03%), *E. cloacae* (1.45%), and *E. sichuanensis* (1.45%)–and eggplant-fed larvae—*E. mundtii* (64.44%), *E. cloacae* (31.11%), *E. ludwigii* (2.22%), and *P. brasiliense* (2.22%). At 37 °C (Figure 4I), three species were isolated from tomato-fed larvae: *E. cancerogenus* (59.72%), *E. mundtii* (38.89%), and *B. wiedmannii* (1.39%). In contrast, eggplant-fed larvae yielded the following five species: *E. mundtii* (68.97%), *E. cloacae* (17.24%), *E. kobei* (6.90%), *E. ludwigii* (3.45%), and *M. luteus* (3.45%).

Overall, these results indicate that the gut microbiota of *T. absoluta* larvae feeding on eggplant exhibits significantly higher species richness and community diversity than those feeding on tomatoes. This trend was consistent across different culture media and incubation temperatures.

## 4. Discussion

In this study, the gut microbes of tomato- and eggplant-fed *T. absoluta* larvae were cultured in three different culture media (namely LB, NA, and BHI) at three different temperatures (25 °C, 31 °C, and 37 °C) to maximize the isolation of cultivable bacteria. Notably, the results showed clear differences in the community structure and diversity of cultivable gut bacteria derived from tomato- and eggplant-fed larvae. Specifically, eight bacterial species were isolated from tomato-fed larvae, whereas eggplant-fed larvae yielded 15 species, with the latter exhibiting significantly greater species richness and unique taxa, such as Actinobacteria (Table 2). These results align with the findings of Yang Yaxian et al. [23], who reported that Bacillota and Pseudomonadota were the dominant phyla in the midgut of *T. absoluta* across different hosts and that the highest gut bacterial diversity was found in eggplant-feeding larvae [23,33]. The results of the present study further confirmed that even if both belong to the Solanaceae family, tomatoes and eggplants differentially influence gut microbial community structures in this insect [34]. This divergence is likely attributable to the differential nutritional composition and secondary metabolites in both plants. Reportedly, *T. absoluta*-infested tomato plants exhibit decreased levels of amino acids, sugars, and defense-related signaling molecules, such as jasmonic and salicylic acids, whereas eggplant maintains a stronger defensive response [4]. Based on this, tomatoes were speculated to provide a nutrient-accessible and low-defense feeding environment, which, in turn, leads to a simplified microbial community structure. This speculation was further corroborated by the absolute dominance of *E. mundtii* and *E. cancerogenus* in tomato-fed populations. In contrast, eggplant exhibits a higher resistance as the host, thereby imposing strong selective pressure on the insect’s gut microbiota through its complex or high-concentration-specific secondary metabolites. This pressure may be attributed to the enrichment of specialized microbial taxa that can survive in the gut environment and potentially assist the host in overcoming plant defenses, as exemplified by the unique presence of *Pseudomonas* and *Pectobacterium* in eggplant-fed populations.

Gut microbiota serves as a crucial “arsenal” for insects against the chemical defenses of host plants [4,35,36]. Herein, *Pseudomonas* species was identified in eggplant-feeding populations. This species has been shown to exhibit detoxification activity in other systems, such as the silkworm–mulberry interactions, where fluorescent *Pseudomonas* degraded the mulberry-specific toxin DNJ [16]. Similarly, *Pantoea* strains, which were isolated from eggplant-feeding larvae, can also contribute to detoxification, as evidenced in the fall armyworm–maize system, where *Pantoea dispersa* metabolized benzoxazinoid compounds and enhanced larval growth [37,38]. Building on this, both *Pseudomonas* and *Pantoea* isolates isolated from eggplant-fed *T. absoluta* were speculated to be involved in neutralizing eggplant-specific defensive metabolites. This microbe-assisted detoxification may be a key mechanism enabling *T. absoluta* to successfully colonize multiple solanaceous hosts. Furthermore, a higher species diversity was observed within the genus *Enterococcus* (including *E. bugandensis*, *E. ludwigii*, and *E. kobei*) in the eggplant-feeding population. *Enterococcus* species generally exhibit a broad carbon utilization capacity and metabolic plasticity, potentially facilitating the metabolism of complex nutritional components in eggplants [39,40,41]. Overall, the structural and functional plasticity of gut microbiota represents a fundamental intrinsic mechanism underlying the adaptability of insects to different host plants [26,42].

Notably, dominant bacteria such as *E. mundtii* remained highly abundant across various conditions in both host populations, suggesting their potential role in sustaining fundamental gut homeostasis. This finding is consistent with the multi-omics findings of Guannan Li et al., who confirmed that *E. mundtii* promotes gut microbial stability in lepidopteran insects by providing a competitive advantage, thereby enabling it to dominate complex microbial environments [43]. Furthermore, *E. mundtii* has been shown to promote insect growth by Li et al. [44], who reported increased larval body length and weight in yellow peach moths. Notably, the functional activities of enterococci in Lepidoptera extend beyond homeostasis and growth. For instance, specific *Enterococcus* strains function as “regulatory hubs,” playing crucial roles in host immune regulation in the diamondback moths [45] while simultaneously elevating their resistance to the insecticide chlorpyrifos and susceptibility to *Bacillus thuringiensis* [46]. Given the pivotal roles of *E. mundtii* and related enterococci in closely related lepidopteran species, it was hypothesized that this bacterium might act as a key regulator in *T. absoluta*, potentially influencing immune responses and mediating host adaptation traits.

We employed a multi-condition cultivation strategy, which revealed the aforementioned differences in microbiota diversity and demonstrated the significant influence of cultivation factors, particularly temperature and medium, on isolated strains. Under identical medium conditions, temperature served as a key environmental factor in shaping the cultivable bacterial community in the guts of *T. absoluta* larvae. Overall, 25 °C was found to be most conducive to maintaining the highest bacterial diversity, with the NA medium yielding the greatest number of species for both host populations. In contrast, specific selective pressure was exerted on microbes at 37 °C, favoring thermotolerant strains, such as *E. mundtii*, which showed significant relative abundance or even became the sole dominant species. Simultaneously, rare strains such as *B. wiedmannii* and *M. luteus*, which were uncultivable at lower temperatures, could also be isolated at 37 °C on the BHI medium. The influence of temperature on the gut community structure observed in this study is consistent with the findings of Chen et al. [47] in the beet armyworm. In both studies, significant shifts in the microbial composition have been noted across temperatures, including elevated abundance of *Enterococcus*. Additionally, the community structure at 31 °C generally represented an intermediate state compared with that in these two extreme conditions. The magnitude of the temperature effect varied with the host plant, with tomato-feeding populations, harboring relatively simple microbial communities, exhibiting greater sensitivity to temperature shifts and a pronounced tendency toward community simplification at elevated temperatures. Conversely, the eggplant-feeding populations, with higher inherent microbial complexity, showed smaller fluctuations in diversity and community structure in response to temperature variations.

At constant temperature, different media simulate distinct nutritional environments. Across all temperature conditions, the NA medium yielded the highest number of bacterial species. For instance, at 25 °C (Figure 4D), seven and nine bacterial species were isolated from the guts of tomato- and eggplant-fed larvae, respectively, which were significantly higher than those in LB or BHI media. Although the number of species on LB medium was few, the genus *Enterococcus*, including *E. mundtii* and *E. cancerogenus*, was markedly enriched, indicating a strong competitive growth advantage under standard nutrient conditions. This genus has also been isolated from other insects, such as the fall armyworm [48] suggesting its conserved role in maintaining gut microbial homeostasis and potential as a bridge in plant–insect–microbe interactions. Nutrient-rich BHI medium supported the isolation of two unique bacterial species, namely *B. wiedmannii* and *M. luteus*. Reportedly, aloe vera juice [49] and citrus peel essential oil extracts [50] have been shown to exhibit antibacterial activity against *M. luteus*, suggesting that specific secondary metabolites in eggplant leaves may similarly regulate the colonization of this bacterium in the insect gut through analogous antimicrobial mechanisms, and thereby participating in host plant–insect–gut microbiota interactions. Collectively, these findings highlight the advantages of employing multi-temperature cultivation strategies for comprehensive analyses of insect gut bacterial diversity and determining both core and specialized functional strains.

The traditional culture methods used in this study, while revealing gut microbiota differences in *T. absoluta* across host plants, are inherently limited. They capture only a minor fraction (<1%) of the microbial community, leaving most uncultured taxa unanalyzed [51,52]. Critically, these uncultured microbes may play vital roles in host ecological adaptation, including functions like detoxification and nutritional complementation not found in culturable isolates [33,53]. For instance, the widespread yet unculturable intracellular symbiont *Wolbachia* has been demonstrated to significantly influence *T. absoluta* population genetics, reproductive biology, and adaptive potential [54,55]. In addition, though three media types and temperature gradients were used, the inherent complexity of the gut microenvironment precludes complete in vivo replication, making it difficult to isolate all microorganisms. We therefore employed diverse nutritional conditions to maximize the recovery of cultivable taxa for downstream analysis [56,57]. Future research needs to focus on the following aspects: (1) the pure strains isolated in this study need to be further analyzed to verify the specific functions of core strains (e.g., *E. mundtii*) [58] and unique strains (e.g., *Pseudomonas* species) in host adaptation using germ-free larval reintroduction experiments [59,60]; (2) metabolomics-based approaches should be integrated to analyze the metabolic differences in identified strains against the chemical profiles of tomato and eggplant leaves in vitro, along with identifying key detoxification or nutritional metabolites; and (3) emerging biological technologies, such as metagenomics, need to be employed to comprehensively explore the functional gene profiles of identified strains.

Altogether, this study employed a multi-condition culture strategy to elucidate the differential effects of different host plants on the gut microbiota of *T. absoluta*. The findings highlight the differences in the species diversity attributable to the feeds derived from host plants. Future studies can elucidate the specific molecular mechanisms underlying plant–insect–microbe interactions [15], providing a foundation for developing novel, sustainable, and precise control strategies against *T. absoluta* [61,62,63].

## Figures and Tables

**Table 1:** Number of bacterial isolates obtained from the larval gut of *Tuta absoluta* under different culture conditions.

Host Plants	Culture Medium	25 °C	31 °C	37 °C
*Solanum lycopersicum*	LB	41	45	38
	NA	88	71	88
	BHI	87	71	88
*Solanum melongena*	LB	58	45	66
	NA	58	41	49
	BHI	31	53	40

LB, Luria–Bertani; NA, nutrient agar; BHI, Brain Heart Infusion.

**Table 2:** Cultural characteristics and cell morphology of culturable gut bacteria from *Tuta absoluta* larvae fed on tomato and eggplant.

Host Plants	Strain NO.	Cultural Characteristics	Cell Morphology	Gram Stain
*Solanum lycopersicum*	T-1	Milky white, circular, smooth, opaque, slightly convex, regular margin	Short rods	-
	T-2	Milky white, circular, smooth, opaque, slightly convex, regular margin	Short rods	-
	T-3	Pale yellow, circular, smooth, opaque, flat, regular margin	Sphericity	-
	T-4	White, circular, smooth, transparent, flat, regular margin	Short rods	-
	T-5	Milky white, circular, smooth, opaque, globose convex, regular margin	Cocci	+
	T-6	Milky white transparent, round, smooth surface, opaque	Cocci	-
	T-7	Milky white, circular, smooth, translucent, flat, regular margin	Short rods	-
	T-8	Milky white, oval, rough, opaque, flat, irregular margin	Long rods	+
*Solanum melongena*	E-1	White, circular, smooth, translucent, flat, regular margin	Short rods	-
	E-2	White, circular, smooth, translucent, flat, regular margin	Short rods	-
	E-3	White, circular, smooth, translucent, flat, regular margin	Short rods	-
	E-4	Yellow, circular, smooth, translucent, flat, regular margin	Short rods	-
	E-5	Milky white, circular, smooth, transparent, flat, regular margin	Long rods	-
	E-6	Pale yellow, circular, smooth, translucent, globose convex, regular margin	Cocci	-
	E-7	White translucent, round, smooth surface, flat, regular edge	Cocci	-
	E-8	Yellow, circular, smooth, opaque, slightly convex, regular margin	Cocci	+
	E-9	Milky white, circular, smooth, opaque, globose convex, regular margin	Short rods	-
	E-10	Milky white, circular, smooth, opaque, slightly convex, regular margin	Short rods	-
	E-11	Milky white, circular, smooth, opaque, slightly convex, regular margin	Short rods	-
	E-12	Yellow, circular, smooth, transparent, flat, regular margin	Cocci	+
	E-13	Yellow, circular, smooth, transparent, slightly convex, regular margin	Short rods	-
	E-14	Milky white, circular, smooth, translucent, flat, regular margin	Long rods	-
	E-15	Milky white, circular, smooth, opaque, globose convex, regular margin	Cocci	+

Note: “+” and “-” indicate Gram-positive and Gram-negative reactions, respectively.

**Table 3:** Comparison of culturable gut bacterial strains from *Tuta absoluta* larvae fed on tomato and eggplant with database-derived reference strains.

Host Plant	Strain No.	Phylum	Family	Genus	The Most Similar Strain	Accession No.	Identity/%
*Solanum* *lycopersicum*	T-2	Pseudomonadota	Enterobacteriaceae	*Enterobacter*	*E.cloacae* strain IRQBAS-34	LT978460.1	99.64
	T-6				*E.sichuanensis* strain XG118	PQ781468.1	99.64
	T-7				*E.cancerogenu* strain XC6	PP471156.1	99.79
	T-4			*Kluyvera*	*K.intermedia* strain F10	MN832981.1	99.79
	T-1		Aeromonadaceae	*Aeromonas*	*A.caviae* strain T25-43	MG737573.1	99.79
	T-3				*A.dhakensis* strain VM30	MF953275.1	99.65
	T-5	Bacillota	Enterococcaceae	*Enterococcus*	*E.mundtii*	KT026102.1	100
	T-8		Bacillaceae	*Bacillus*	*B.wiedmannii* strain ER6	MT124531.1	99.80
*Solanum melongena*	E-7	Pseudomonadota	Enterobacteriaceae	*Enterobacter*	*E.bugandensis* strain PxG11	MN883866.1	99.72
	E-9				*E.ludwigii* strain HE15	LN624804.2	99.86
	E-11				*E.kobei* strain NG-2	PX022382.1	99.72
	E-10				*E.cloacae* strain NP07	MH169128.1	99.79
	E-1		Pectobacteriaceae	*Pectobacterium*	*P.Brasiliense* strain 1b	MT240621.1	99.78
	E-2				*P.carotovorum* strain G-1-5-1	EF102836.1	99.78
	E-3				*P.aroidearum* strain M5162	PV596576.1	99.72
	E-6		Erwiniaceae	Pantoea	*P.eucrina* strain CX-112	MH368427.1	99.57
	E-4		Pseudomonadaceae	*Pseudomonas*	*P.oryzihabitans* strain OsEnb_ALM_C2	MN889340.1	99.86
	E-5	Bacillota	Bacillaceae	*Mesobacillus*	*M.thioparans* strain KmS3200909	MG011570.1	99.79
	E-14			*Bacillus*	*B.senegalensis* strain RS8	AF519468.1	99.36
	E-15		Enterococcaceae	*Enterococcus*	*E.mundtii* strain NM-1	KT722998.1	100
	E-8		Staphylococcaceae	*Mammaliicoccus*	*M.sciuri* strain X302	KU240493.1	99.79
	E-12	Actinomycetota	Micrococcaceae	*Micrococcus*	*M.luteus* strain 2-13-826-2	OP108647.1	99.21
	E-13		Dermabacteraceae	*Brachybacterium*	*B.paraconglomeratum* strain ER41	MT124566.1	100

## Data Availability

The original contributions presented in this study are included in the article. Further inquiries can be directed to the corresponding authors.

## Abbreviations

The following abbreviations are used in this manuscript:

LB	Luria–Bertani
NA	nutrient agar
BHI	Brain Heart Infusion
PCR	polymerase chain reaction
DNJ	1-deoxynojirimycin
